# Mechanistic Effects of Calcitriol in Cancer Biology

**DOI:** 10.3390/nu7065020

**Published:** 2015-06-19

**Authors:** Lorenza Díaz, Mauricio Díaz-Muñoz, Ana Cristina García-Gaytán, Isabel Méndez

**Affiliations:** 1 Departamento de Biología de la Reproducción, Instituto Nacional de Ciencias Médicas y Nutrición Salvador Zubirán, Vasco de Quiroga No. 15, Tlalpan, Mexico City 14000, Mexico; E-Mail: lorenzadiaz@gmail.com; 2 Departamento de Neurobiología Celular y Molecular, Instituto de Neurobiología, Universidad Nacional Autónoma de México (UNAM), Campus UNAM-Juriquilla, Blvd. Juriquilla 3001, Querétaro 76230, Mexico; E-Mails: mdiaz@comunidad.unam.mx (M.D.-M.); acgarciag02@gmail.com (A.C.G.-G.)

**Keywords:** calcitriol, cancer, inflammation, vitamin D deficiency, oncogenesis

## Abstract

Besides its classical biological effects on calcium and phosphorus homeostasis, calcitriol, the active vitamin D metabolite, has a broad variety of actions including anticancer effects that are mediated either transcriptionally and/or via non-genomic pathways. In the context of cancer, calcitriol regulates the cell cycle, induces apoptosis, promotes cell differentiation and acts as anti-inflammatory factor within the tumor microenvironment. In this review, we address the different mechanisms of action involved in the antineoplastic effects of calcitriol.

## 1. Vitamin D. General Concepts

The classical function of vitamin D is to maintain calcium and phosphorous homeostasis and consequently preserve bone health. Its deficiency was first described related to rickets at the beginning of 20th century; however, since the 19th century people knew that rickets could be prevented by sun exposure and consuming fish oil [1]. Vitamin D helps to regulate calcium and phosphorus serum concentrations. Its classical hormonal actions take place in the kidney, intestines and bones [2].

Two main forms of vitamin D are present in nature: vitamin D_2_ or ergocalciferol, which is obtained from fungi and vitamin D_3_ or cholecalciferol, which is biosynthesized in the skin by sun ultraviolet B (UVB) radiation exposure. The latter is the most important source of vitamin D, but it may also be obtained from the diet; for instance, high levels of vitamin D may be found in oily fish and egg yolk, or, alternatively, obtained by vitamin D supplementation. Biosynthesis of cholecalciferol begins with the non-enzymatic conversion from 7-dehydrocholesterol to precholecalciferol-7 (previtamin-D_3_), which in turn is rapidly converted to cholecalciferol at body temperature [3]. Cholecalciferol is transported to the liver where it is hydroxylated by 25-hydroxylase to form 25-hydroxyvitamin D, also called calcidiol (25OHD), the most abundant and stable vitamin D metabolite. Calcidiol is hydroxylated by 1-α-hydroxylase to 1,25-dihydroxyvitamin D or calcitriol (1,25(OH)_2_D) [4], the most active vitamin D metabolite. The enzyme 1-α-hydroxylase is the cytochrome p450 27B1 (CYP27B1), encoded by the *CYP27B1* gene in humans, and is present in the kidneys and in other extrarenal sites including tumors [5]. Calcidiol and calcitriol are transported in serum bound to the vitamin D-binding protein (DBP) and are ultimately transformed to more soluble compounds by 24-hydroxylase. This enzyme catalyzes the degradation of calcitriol to metabolites that are excreted in bile, feces and urine [6,7]. Synthesis, catabolism and excretion determine the blood concentrations of vitamin D. Calcidiol is the form of vitamin D of clinical importance to evaluate vitamin D sufficiency since it is the most abundant and stable vitamin D metabolite and correlates with total vitamin D exposure from both endogenous production and the diet [8]. There are factors or conditions that stimulate calcitriol synthesis such as parathyroid hormone (PTH), hypocalcemia and hypophosphatemia, all of which up-regulate CYP27B1.

Calcitriol actions as a steroid hormone are mediated through the vitamin D receptor (VDR) [9], which is a high affinity ligand-activated transcription factor. In the classic genomic mechanism of action of calcitriol, activated VDR heterodimerizes with the retinoid X receptor (RXR); this complex binds to vitamin D response elements (VDREs) in the promoter of target genes and recruits co-activators and co-repressors to induce or inhibit gene transcription [10]. Some genes that contain VDREs in their promoters are osteocalcin, calcium binding protein and 24-hydroxylase. Classical effects of vitamin D comprise regulation of calcium and phosphate homeostasis and proper bone mineralization, working in concert with PTH. Calcitriol acts on kidney, intestine, parathyroid gland and bone through non-genomic and genomic pathways to achieve mineral homeostasis [11].

Additionally, calcitriol may behave as a first messenger and exert non-genomic effects that involve membrane receptors such as the classic VDR localized at the membrane and the disulfide isomerase-associated 3 (PDIA3) protein, also known as membrane-associated rapid response steroid specific (MARRS). By interacting with these receptors calcitriol may rapidly initiate the synthesis of second messengers such as cyclic AMP (cAMP), cGMP, inositol triphosphate, diacylglycerol and calcium [12,13,14,15,16,17,18]. Then, the signals of these small molecules may be amplified by the consequent activation of cascades of downstream proteins or secondary effectors such as protein kinase A (PKA), protein kinase C (PKC), mitogen activated protein kinase (MAPK) and calmodulin (CaM) [18,19,20]. The activation of these signaling pathways can also result in the rapid opening of ion channels, such as the voltage-gated Ca^2+^ channel from the L-type [21]. Both membrane and nuclear VDRs may participate in the final effects of calcitriol in different types of cells; for instance, in prostate cancer cells [22] and muscle cells [23,24]. Interestingly, the induction of the calcitriol degrading enzyme CYP24A1 by calcitriol also seems to be the result of cooperation between genomic as well as non-genomic regulation. On one hand, calcitriol potently upregulates CYP24A1 through the well-known VDREs located in its promoter sequence [25,26]. On the other hand, a functional Ets-1 binding site downstream of the proximal VDRE (–128/–129) has been shown to be very important to achieve maximal calcitriol inductive capacity of the CYP24A1 promoter. The calcitriol-dependent activation of Ets-1 involved the phosphorylation of Ets-1 by the MAPK ERK5; thus, highlighting the relationship between the non-genomic and the genomic activation of the CYP24A1 promoter by calcitriol [27,28]. In addition, it was shown that the phorbol ester 12-myristate 13-acetate (PMA) had the capacity to potentiate the stimulatory effect of calcitriol upon CYP24A1 gene expression in a PKC dependent manner in rat renal and intestinal epithelial cells [29,30,31]. Also, a vitamin D stimulatory element upstream of the VDRE1 (–171/–163) has been described and it was shown to be responsible for the synergy between PMA and calcitriol, in a process dependent on ERK1/2 and c-Jun N-terminal kinase (JNK) activities in renal cells [32]. Of note, the cross-talk between the genomic and non-genomic signaling pathways upon the cytochromes involved in vitamin D metabolism has proven to be tissue specific. For instance, whereas in the kidney PTH causes the upregulation of CYP27B1 gene expression through a cAMP-dependent process, in the human placenta this second messenger exerts the opposite effect [33,34]. Indeed, in the placenta, evidence has been provided showing that calcitriol and cAMP downregulate CYP27B1 by a PKA signaling pathway, probably through the cAMP response element (CRE) that is present in its promoter, and independently of the VDR. In fact, the PKA selective inhibitor H-89 but not a VDR antagonist prevented the calcitriol-mediated CYP27B1 gene expression inhibition [34]. In line with these observations, the incubation of syncytiotrophoblast cells from human placentas in the presence of calcitriol showed increased intracellular content of cAMP [15].

In normal cells such as those of colon, intestine, bone and muscle, the calcitriol-dependent activation of some kinases cascades and the rise in intracellular calcium may promote beneficial proliferative effects [16,18,20,35]. For instance, it could represent a potential mechanism by which calcitriol affects colonocyte growth and maturation, as previously suggested by Ramesh *et al.* [16]. Indeed, it was demonstrated that *in vivo* treatment of rats with calcitriol resulted in 44% more [^3^H]thymidine/mg of DNA incorporation in rat colonocytes than vehicle-treated animals [16]. Similar results were obtained in intestinal mucosal cells, where the vitamin D-dependent stimulation of mucosal cell proliferation preceded enhanced calcium transport, suggesting that this phenomenon may be an important component of calcitriol action on the intestine [35]. However; in the context of cancer, activation of these mitogenic pathways may not be so appealing. Regarding this, Wang and colleagues recently demonstrated that the calcitriol-dependent stimulation of the MAPK ERK5 pathway diminishes the ability of calcitriol to induce terminal differentiation of human myeloid leukemia cells. This was a consequence of the ability of calcitriol to activate the proto-oncogene kinase Cot1, resulting in the upregulation of ERK5. Interestingly, the authors showed that the pharmacological inhibition of MAPK activity by XMD8-92 enhanced the calcitriol-induced G1 and G2 cell cycle arrest of leukemia cells, strongly suggesting that the combination of calcitriol with ERK5 inhibitors may be more successful in cancer treatment than calcitriol alone [36]. Therefore, it is imperative to correctly understand the mechanistic effects of calcitriol in order to fully take advantage of its anti-neoplastic effects in the clinic. Herein we present an in-depth review of the different pathways and mechanisms of action of calcitriol in the context of cancer biology.

## 2. Vitamin D and Cancer

### 2.1. Biochemical Basis of Cancer

Cancer cells show a set of characteristics that make them capable of uncontrolled proliferation, surviving by avoiding programmed cell death, and using lactate as principal metabolic fuel [37]. In recent years, a set of 10 hallmarks have been proposed to delineate the multistep process that leads to neoplastic transformation of normal cells. They include genome instability, avoiding immune attack, evading growth suppressors, enabling replicative immortality, resisting cell death, tumor-promoting inflammation, sustaining proliferative signaling, inducing angiogenesis, activating invasion and metastasis, and deregulating cellular energetics [38]. From a biochemical point of view, the main metabolic adaptation of tumor cells is an increased rate of aerobic glycolysis, known as the Warbug effect [39]. This effect, described almost a century ago, is a diminution in mitochondrial activity in spite of adequate O_2_ availability. As a consequence, the principal metabolic output of glucose, the production of lactate, is enhanced. It has been suggested that one important aspect of the Warburg effect in cancerous cells is to produce biomass (lipid, protein, and nucleotide) that can sustain the proliferative capacity of the rapidly dividing cells [40].

In many cases tumorigenesis is preceded by episodes of chronic inflammation caused by a variety of environmental factors (e.g., tobacco, asbestos, ozone, *etc.*). This pro-oxidant response promotes an elevation of reactive oxygen species (ROS) and reactive nitrogen intermediates (RNI). The increased production of ROS and RNI takes place in different cellular compartments, but primarily within the mitochondria [41]. The redox dysregulation eventually modifies the signaling pathways associated with factors such as the nuclear factor kappa-light-chain-enhancer of activated B cells (NFκB), hypoxia-inducible factors (HIFs), phosphoinositol-3 kinase/protein kinase B (PI3K/AKT), and others. These alterations in the signaling of the affected cells promote the synthesis and secretion of chemokines, cytokines and prostaglandins related to the onset of neoplasia [41].

### 2.2. Deficiency of Vitamin D in Cancer

In *in vitro* and *in vivo* animal models, calcitriol has been shown to have anti-proliferative, pro-differentiative and pro-apoptotic actions in cancer cells. In this manner, vitamin D could limit cancer progression or prevent it. A large number of observational studies have shown that low circulating levels of cholecalciferol, which are related to geographical location, diet and activity, are associated with a higher risk of cancer and cancer-specific worse prognostic. However, data regarding the role of vitamin D in cancer risk, incidence and mortality is still controversial. Some studies suggest a positive correlation between circulating 25OHD concentrations in patients with a diagnostic of cancer, although several epidemiologic studies including colorectal, breast, and hepatocellular carcinoma have demonstrated an inverse association between serum 25OHD levels and the risk to develop these pathologies. It has not yet been clearly elucidated if low serum 25OHD levels are causative of associative parameters of cancer. Some evidences are described below.

Colorectal cancer is the third most commonly diagnosed cancer in both men and women. Colorectal cells express VDR and 1-α-hydroxylase, which suggests a role of vitamin D in the development of this cancer. Indeed, in a meta-analysis of eight prospective studies, an inverse association between circulating 25OHD serum levels and colorectal cancer, with a stronger association for rectal cancer, has been observed [42]. In line with this research, in a study with a cohort of 25,000 men and women, an association between high pre-diagnostic serum levels of 25OHD and reduced risk of colon cancer was found [43]. These observations suggest that low levels of vitamin D are related to increased risk of colorectal cancer.

Breast cancer is the most frequent form of cancer in women and it is the leading cause of female cancer death worldwide [44]. Breast tumors are highly heterogeneous and are classified on the basis of the expression of estrogen receptors (ER), human epidermal growth factor receptor 2 (HER2) and progesterone receptors (PR), or the gene expression profiles as luminal or basal [45]. Approximately 30% to 50% of subjects with early-stage breast cancer progress to metastatic stage. Cancer metastasis involves a cascade of events including extravasation and outgrowth of disseminated cells that undergo epithelial-mesenchymal transition at a secondary site. Acquired resistance to chemotherapy represents a major clinical obstacle in successful treatment of breast cancer. Breast epithelial cells contain the enzymatic machinery for vitamin D metabolism, as well as VDR. However, the association between serum 25OHD concentrations and breast cancer risk is controversial. Some studies have not found an association between circulating 25OHD concentrations and overall breast cancer risk, while others have reported an inverse association between circulating 25OHD concentrations and breast cancer risk or a moderate association of high levels of 25OHD, and perhaps 1,25(OH)_2_D, with reduced risk of breast cancer [46,47]. There is limited evidence for an association of vitamin D with outcomes in breast cancer survivors. In a breast cancer cohort, higher serum 25OHD levels were associated with overall but not breast cancer-specific survival [48]. Also, in a study that examined calcium plus vitamin D supplementation on breast cancer suggested a decrease in breast cancer risk [49]. It remains unclear whether hypovitaminosis D is a causality or effect of breast cancer development, as well as which are the optimal serum 25OHD levels to reduce breast cancer risk. Nevertheless, the fact that serum levels of 25OHD of 52 ng/mL have been associated with a 50% reduction of the risk to develop breast cancer compared to women with 25OHD <13 ng/mL, highly suggests avoiding vitamin D deficiency/insufficiency in order to easily and economically prevent this pathology [50,51]. From this point of view, hypovitaminosis D is one risk factor for breast cancer that can be prevented and modified either by supplementation or sun exposure.

Hepatocellular carcinoma (HCC) is the sixth most common neoplasia and the third most common cause of cancer death worldwide [52]. The main risk factor for HCC is chronic infection with hepatitis B or C that may lead to cirrhosis which is present in 80% to 90% of the cases of HCC [53]. Hepatocarcinogenesis is a complex multistep histological process with a heterogeneous molecular profile that involves many modified signaling pathways. These include deregulation of tumor suppressor genes and oncogenes such as TP53, present in about 40% of cancers, CTNNB1 (β catenin gene), expressed in about 25% of HCC due to hepatitis C virus, ErbB tyrosine kinase receptor family members, E-cadherin and cyclooxygenase 2 (COX-2) [53,54]. Epigenetic events occurring during the development of this pathology have been reported in HCC, such as aberrant methylation patterns in E-cadherin, COX-2, and p16 genes, among others [55,56]. The expression of p16 and COX-2 has been associated with inhibition of cell proliferation; while hypermethylation silences expression of these genes. Vitamin D deficiency has been associated with HCC [57,58], and a causal relationship of low vitamin D levels with HCC development has been proposed [59]. Most European patients with chronic liver diseases suffer from osteopenia and osteoporosis [58]. In a randomized trial, postmenopausal women that presented a high fracture incidence and low concentrations of calcium and vitamin D were treated with calcium plus vitamin D. This approach resulted in lower cancer incidence in the treated group than in placebo control subjects [60]. The anti-fibrotic effect of vitamin D involves regulating activation of stellate cells and reducing Smad3 occupancy on the promoter of pro-fibrotic genes [61], suggesting a permissive role of vitamin D that favors the quiescent state of non-activated stellate cells in the physiology of the liver. However, as in the other types of cancer discussed herein, in HCC it is not clear if vitamin deficiency is a cause or a consequence of the pathology. In a genetic analysis, patients with hepatitis C virus-associated HCC showed correlation between reduced 25OHD serum levels and the presence of single nucleotide polymorphisms (SNPs) in GC (encoding the vitamin D binding protein), DHCR7 (encoding 7-dehydrocholesterol reductase) and CYP2R1 (encoding a liver 25-hydroxylase) [62], that are genetic determinants of vitamin D serum levels. In contrast, no association was found between these genetic variations and liver fibrosis progression rate. The authors concluded that there is a relatively weak but functionally relevant role for vitamin D in the prevention of HCV-related hepatocarcinogenesis [62].

To date, the effects of vitamin D on carcinogenesis or cancer progression remains incompletely understood. However, a great body of information about the association between vitamin D deficiency and increased cancer risk, together with the known benefits of maintaining adequate 25OHD serum levels for cancer prevention as well as the therapeutic potential of calcitriol in the oncology field, warrant further investigations of this natural antineoplastic hormone. Because of the hypercalcemic effects of calcitriol, recent research has focused on the use of calcitriol analogs that have more potent anti-proliferative effects on cancer cells and fewer calcemic side effects.

## 3. Mechanistic Effects of Calcitriol in Cancer Prevention and Treatment

**Figure 1:**
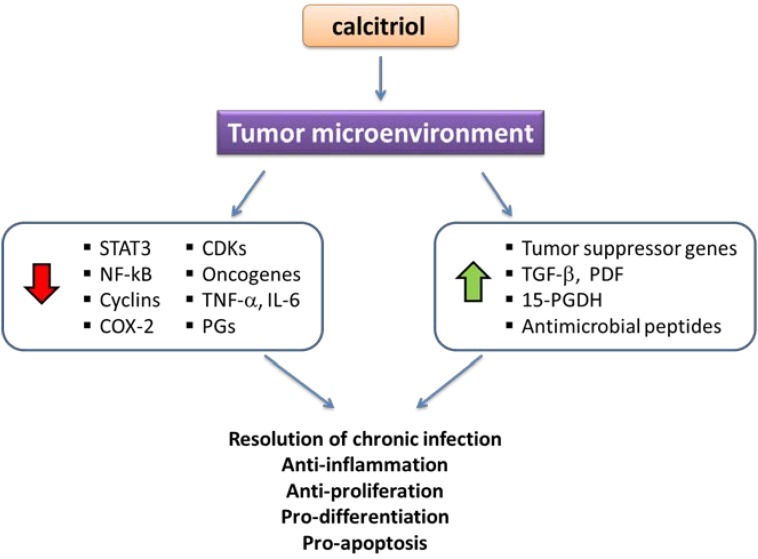
Calcitriol effects in the tumor microenvironment. Calcitriol, either activated from 25OHD in the kidney or intratumorally, acts up-regulating (green arrow) or down-regulating (red arrow) different factors that result in the indicated final biological effects. PGs, prostaglandins; 15-PGDH, 15-hydroxyprostaglandin dehydrogenase; COX-2, cyclooxygenase-2; TNF-α, tumor necrosis factor-α; IL-6, interleukin 6; PDF, prostate deriver factor.

It is now well recognized that non-calcemic effects of calcitriol are as important as the classic ones. Indeed, calcitriol not only modulates calcemia and phosphatemia, but also affects the functions of organs and systems in a paracrine and/or autocrine manner. In particular, a vast amount of epidemiologic and clinical evidence has uncovered the close relationship that exists between vitamin D deficiency and the risk of cancer development. For instance, it has been postulated that more than 220,000 new cases of breast and colorectal cancer would be prevented annually worldwide simply by raising serum calcidiol concentrations to approximately 40–60 ng/mL [63]. Also the incidence of cancer tumors is lower in latitudes with high solar exposure [64,65,66,67]. What are the molecular bases for these oncoprotecting effects of vitamin D? As previously discussed, calcidiol may be activated extrarenally in different tissues that express CYP27B1, like the breast, ovaries, lung, kidney, stomach and tumor-derived cells as well [68,69,70]. When activated by CYP27B1, calcidiol is transformed into calcitriol, which, acting mainly through the VDR, exerts both autocrine and paracrine anti-cancer activity (Figure 1). Herein, we will review various mechanisms of action of calcitriol involved in its anti-neoplastic effects, which explain why this secosteroid stands as a prominent endogenous natural cancer preventing factor and promising therapeutic agent. It is noteworthy that the molecular mechanisms herein described may be exerted differentially by calcitriol depending on the tissue, and that not all cells respond to calcitriol in the same manner. In general, the expression and functionality of the VDR is mandatory for the anticancer effects of calcitriol to take place; therefore, the loss of this transcription factor, as seen in some cells after malignant transformation, results in calcitriol resistance.

### 3.1. Calcitriol and Anti-Proliferative Effects

Perhaps the most recognized anti-neoplastic effect of calcitriol is its ability to inhibit cell proliferation. One of the first demonstrations of a calcitriol-mediated action on tumor cells was performed by Colston and Collegues in 1981, who showed that incubation of malignant melanoma in the presence of calcitriol inhibited cell proliferation, producing a marked increase in cell doubling time [71]. From that date on, numerous studies have corroborated the anti-proliferative activity of calcitriol in a wide variety of normal and cancerous cells, gaining the attention of researchers and clinicians in the oncology field. Cell proliferation requires fluent transition of cells through a sequence of events leading to their replication. This process must be highly regulated, because its dysregulation may result in pathological events such as cancer. Indeed, cell cycle progression is positively and cooperatively regulated by cyclins and CDKs. The catalytic activity of CDKs, in turn, is negatively controlled by CDK-inhibitors (CKI) such as the members of the Cip/Kip family, also known as tumor suppressor genes. Among the members of this family are p21 and p27, implicated in promoting the G0/G1 cell cycle arrest [72]. Some cell cycle regulatory factors are targets of calcitriol either directly or indirectly through VDREs in their promoter regions. For instance, in squamous carcinoma cell lines of the head and neck, the cell-cycle inhibitor protein p21 was identified as a direct target of calcitriol, inducing G0/G1 cell-cycle arrest [73]. Moreover, in breast cancer cells at least three functional VDREs were located in the promoter of p21 [74]. Similarly, marked up-regulation of p21 and p27 expression and significant down-regulation of cyclins and CDKs after calcitriol treatment were reported in pancreatic cancer cell lines [75]. These early events were related to the consequent block of the G1/S transition and growth inhibition; and were also observed in other cancer cells such as mammary, prostate and multipotent mesenchymal cells [76,77,78]. In these multipotent cells, calcitriol blocked the transition from G1 to S phase of the cell cycle, and cells accumulated in G1 phase due to reduced expression of the cyclins A2, B1, B2, D1, D3, E1 and F, as well as the CDKs Cdk2 and Cdk4 [77]. This effect was accompanied by increased expression of p21, as occurred in other cell types, which is relevant considering that the transcriptional activation of p21 by calcitriol has been shown to initiate the differentiation process, leading to a terminal differentiation program [79]. Nevertheless, in squamous carcinoma, even though calcitriol increased p27 inhibition of cell growth *in vivo* and *in vitro*, it downregulated p21 [80], suggesting that calcitriol inhibits cell proliferation using different mechanisms in each cell type. Interestingly, calcitriol may act synergistically with other antineoplastic drugs currently used in the clinic. In this regard, clinical and preclinical studies have demonstrated that calcitriol or its analogs potentiate the therapeutic effect of taxanes, platinum analogues, alkylating agents, tyrosine kinase inhibitors and ionizing radiation [81,82,83,84,85,86]. On the other hand, calcitriol can also induce other tumor suppressor genes, including BRCA1, p53 and the p53 homologue p73 [83,87,88,89]. The fact that calcitriol stimulates p53 expression is crucial in the context of cancer, given that p53 is considered the “guardian of the genome” due to its ability to halt the cell cycle when abnormal mitotic events occur. In addition, calcitriol indirectly affects the product of another important tumor suppressor gene, namely, retinoblastoma (Rb). Rb prevents unnecessary cell growth by inhibiting cell cycle progression through sequestering the transcription factor E2F. If Rb becomes phosphorylated (pRb), for example by certain CDKs or oncogenic proteins such as E6 from human papilloma virus, it becomes inactive liberating E2F and thus, allowing cell cycle progression to proceed. Calcitriol markedly prevents Rb phosphorylation while it promotes the dephosphorylation of pRb, contributing in this manner to the inhibition of malignant cell proliferation [80,90,91].

### 3.2. Calcitriol Pro-Differentiating Effects

The process of carcinogenesis is, in a way, the opposite of what happens during morphogenesis. In the latter, cells become specialized in order to form specific organs and tissues; while in the former, transformed cells undergo a dedifferentiating process, losing their acquired specialized phenotype while gaining the ability to proliferate indefinitely. Calcitriol is a well-known prodifferentiating hormone which regulates the activity of more than 60 genes involved in cell differentiation. For example, in colon carcinoma calcitriol and its nonhypercalcemic analogues promote differentiation by inhibiting β-catenin signaling and inducing the expression of adhesion proteins such as E-cadherin, occludin, and vinculin [92]. These effects are potentially therapeutic, given that adhesion proteins maintain cell bonding, favoring tissue integrity; therefore, while their suppression is associated with loss of contact inhibition, tumorigenesis, and metastasis, their induction is considered to be beneficial. Moreover, the VDR binds to β-catenin inactivating it, precluding in this manner β-catenin-mediated oncogenic processes [92]. Colonic tumorigenesis is a gradual process that may last as long as 20 years during which aberrant crypt foci are formed while genetic and epigenetic mutations accumulate, resulting in the loss of the epithelial phenotype which includes E-cadherin expression. Interestingly, epidemiological data suggest that 25OHD serum levels inversely correlate with the risk of colon cancer development, which highlights the importance of the calcitriol-dependent E-cadherin induction [93,94]. Another example of calcitriol pro-differentiating effects has recently been described in estrogen receptor α (ERα)-negative breast cancer cells. In these cells, calcitriol, acting through the VDR, was able to induce the expression of a functional ERα. Most important, the calcitriol-induced ERα restored the response to anti-estrogens such as fulvestrant and tamoxifen, allowing these compounds to inhibit cell proliferation as they do in ERα-responsive cells [95]. These results may be applicable as a therapeutic alternative, particularly in those patients affected with ER-negative tumors, by enhancing their sensitivity to hormonal therapy.

### 3.3. Apoptosis

The process of apoptosis is genetically programmed cell death. Calcitriol is able to modulate apoptosis mediators by diverse mechanisms that favor the elimination of malignant cells. Indeed, calcitriol represses the anti-apoptotic proteins Bcl-2 and Bcl-X_L_ while stimulates the pro-apoptotic proteins Bax, Bak, and Bad in various cancer cells [96,97,98,99,100]. Moreover, calcitriol induces activation of caspases, either alone or combined with standard chemotherapeutic drugs, such as gemcitabine, the agent used to treat pancreatic cancer [101,102]. Once the caspase signaling cascade has been activated, the cell is irretrievably committed to apoptosis. As mentioned before, calcitriol can be used in combination with current chemotherapeutic drugs; for example, in epidermal growth factor receptor (EGFR)- and HER2-positive breast cancer cells, the synergistic anti-proliferative effect of the combination of gefitinib with calcitriol or its synthetic analogs was due to the induction of apoptosis mediated by the upregulation of the pro-apoptotic protein BIM and activation of caspase 3 [86]. Calcitriol may also induce cell death by alternative pathways, such as increasing the calcium concentration, releasing cytochrome *c* and reducing intracellular glutathione, which results in the production of ROS [103,104,105]. Glutathione is an anti-oxidant that protects the cells from ROS; therefore, calcitriol-dependent glutathione reduction favors apoptosis. In human breast cancer cells, calcitriol enhanced the H_2_O_2_-dependent activation of caspase-7 while induced caspase-independent cell death by releasing cytochrome *c* [104]. Interestingly, calcitriol effects may be potentiated by the hormonal milieu, as observed in endometrial cancer cells, in which incubation with both progesterone and calcitriol upregulated the expression of Bax and other apoptosis-related proteins, inhibiting endometrial cancer cell growth by inducing apoptosis and cell cycle arrest [106].

Telomerase inhibition is under active investigation as an oncologic target. The enzyme telomerase adds DNA sequence repeats to the DNA strands in the telomere regions located at the ends of eukaryotic chromosomes, but it is naturally repressed in mature somatic cells. However, cancerous cells aberrantly profit from this process in order to become immortal. Accordingly, telomerase activation has been observed in ~90% of all human tumors [107]. This could imply that telomerase repression in tumor cells may result in growth inhibition. Interestingly, in ovarian cancer and prostate cancer cells, calcitriol transcriptionally represses the catalytic subunit of telomerase, namely, telomerase reverse transcriptase (TERT), which results in decreased telomerase activity [108,109]. Similarly, during calcitriol-dependent induction of differentiation in leukemia cells, the expression of TERT mRNA is inhibited as well [110]. Alternatively, calcitriol may also suppress TERT expression indirectly by inducing micro-RNA (miRNA). For instance, miRNA-498 has been recently described as a novel target of calcitriol that decreases TERT expression, induces cell death, and suppresses tumor growth [111]. A functional VDRE was found in the 5-prime regulatory region of the miRNA-498 genome. This miRNA targeted the 3-prime untranslated region of human TERT mRNA, decreasing its expression [111].

### 3.4. Regulation of Oncogenes

For malignant transformation to occur, a number of mutations must take place in the cell. Generally, these mutations arise in tumor suppressor genes and proto-oncogenes. Proto-oncogenes are normal genes that become oncogenes due to mutations or increased expression. Regarding the effects of calcitriol upon oncogene regulation, it is widely accepted that this hormone transcriptionally represses a variety of oncogenes which may differ according to the type of tumor. For example, in tumoral cells from breast cancer (MCF-7, T-47D) and ovarian carcinoma (NIH:OVCAR3) calcitriol inhibited the expression of *c-myc* protein more than 50% compared to controls [112]. Calcitriol also provoked a 50% decrease in *c-myc* mRNA but a much greater reduction of *c-myc* protein in prostate cancer cells, which resulted in growth inhibition [113]. These findings are relevant considering that the inactivation of *c-myc* has shown to halt tumor progression, since this oncogene is a positive regulator of the transcription factor E2F, implicated in cell proliferation. Indeed, studies in HCC showed that even the transient inactivation of *c-myc* completely reversed tumorigenesis, reprogramming the malignant cells into normal cellular lineages [114,115]. Accordingly, although the regulation of *c-myc* was not tested, in a phase II study undertaken in patients with inoperable HCC, the administration of the vitamin D analogue Seocalcitol reduced tumor dimensions in some patients, demonstrating the *in vivo* anti-neoplastic activity of this vitamin D analogue [116]. Another oncogene that is transcriptionally regulated by calcitriol is *c-fos*, which is implicated in the regulation of cell growth. Both *c-myc* and *c-fos* bear VDREs in their promoter regions [117,118,119], rendering them susceptible for calcitriol regulation. A prominent oncogene involved in carcinogenesis of multiple cell types is KCNH1, which codes for the voltage-gated potassium channel ether *à go-go* 1 (Eag1) [120]. Overexpression of KCNH1 confers loss of contact inhibition and favors tumor cell proliferation, while its genetic or pharmacological inhibition blunts cancer cell growth. In this regard, evidence has been provided showing that the expression of KCNH1 is significantly inhibited by calcitriol *in vitro* in different types of breast and cervical cancer cells and *in vivo* in human breast cancer tumors xenografted in athymic mice [121,122,123]. In these studies the calcitriol-dependent downregulation of Eag1 was accompanied by inhibition of cell proliferation and reduction of tumor growth. Recently, proof of the regulatory mechanism involved in the repressive effect of calcitriol upon the oncogene KCNH1 has been provided [124]. Indeed, a functional negative VDRE E-box type in the hEAG1 promoter has just been described [124]. These studies highlight the potential therapeutic effect of calcitriol in VDR-positive tumors that overexpress Eag1.

### 3.5. Regulation of Angiogenesis

In the case of cancer, angiogenesis is one of the least desirable hallmarks as it suggests a poor prognosis, rapid tumor development and metastasis. Indeed, tumor vascularization is needed for growth and dissemination. Initiation of angiogenesis is triggered when anti-angiogenic factors are overridden by pro-angiogenic factors, whose production may be regulated by oncogenes and tumor suppressor genes. Vascular endothelial growth factor (VEGF) is considered the master pro-angiogenic factor, while Thrombospondin-1 (Tsp-1) is usually recognized as an anti-angiogenic molecule [125,126]. There is a great deal of evidence showing that calcitriol inhibits angiogenesis both *in vitro* and *in vivo*. For example, calcitriol has been shown to inhibit the growth of tumor-derived endothelial cells (TDECs) [127], while in an *in vivo* preclinical model of VDR knockout mice, increased levels of HIF-1α, VEGF, angiopoietin 1 and platelet-derived growth factor were observed compared to control mice, suggesting that calcitriol-mediated anti-proliferative effects on TDEC are VDR-dependent [128]. Similarly, in 3D collagen gels, calcitriol inhibited endothelial cell sprouting and morphogenesis and had a small, but significant, inhibitory effect on VEGF-induced endothelial cell proliferation [129]. *In vivo*, VEGF-dependent tumor growth was significantly inhibited by calcitriol administration to mice xenografted with MCF-7 breast cancer cells that over-expressed VEGF [129]. Likewise, in X-ray immunosuppressed BALB/c mice xenografted with human tumor cell lines of different origin, systemic treatment with calcitriol significantly decreased angiogenesis [130], while immunohistochemical analysis of colon tumors from calcitriol-treated rats demonstrated a significant decrease in VEGF expression and microvessel counts compared to tumors from untreated animals [131]. Nevertheless, there is also compelling evidence that calcitriol can significantly induce VEGF expression and secretion, thereby stimulating vascular cell proliferation [132,133,134,135,136,137]. In fact, calcitriol may either induce or inhibit angiogenesis, depending on the context and cell type. By instance, human dendritic cells (DC) treated with calcitriol secreted large amounts of VEGF, while non-treated cells remained inactive [135]. This calcitriol-induced VEGF was able to elicit a marked angiogenic response (which was inhibited by neutralizing anti-VEGF antibodies or by a VEGF receptor-2 inhibitor) when implanted in the chick embryo chorioallantoic membrane. In the context of cancer, incubation of a large panel of breast cancer cells with different molecular signatures in the presence of calcitriol significantly inhibited Tsp-1 but stimulated VEGF mRNA expression and protein secretion [138], suggesting the induction of a pro-angiogenic phenotype. Accordingly, the *in vivo* administration of calcitriol resulted in significantly elevated serum VEGF levels and undetectable Tsp-1 in breast cancer-xenograft-bearing mice, compared to untreated controls. This, however, did not abrogate the inhibitory effect of calcitriol on tumor growth, indicating that the anti-neoplastic activity of calcitriol *in vivo* involves different mechanisms that are not necessarily related to the inhibition of tumor vascularization [138]. In accordance with this study, different *in vivo* models of prostate cancer calcitriol and several of its less calcemic analogues did not inhibit angiogenesis, despite showing tumor growth inhibition [139]. The authors suggested that VDR-dependent anti-proliferative effects, and not the inhibition of angiogenesis, were the main mechanisms of action of calcitriol and its analogues [139]. Most probably, the ultimate effect of the secosteroid hormone upon angiogenesis is related to the equilibrium between pro-and anti-angiogenic molecules, as seen in cells of human colon carcinoma, where VEGF and Tsp-1 promoter activity, as well as their mRNA expression were stimulated in a similar manner by calcitriol, [132]. Interestingly, the concomitant expression of both factors was needed to restrain angiogenesis, since Tsp-1 neutralizing antibodies shifted the angiogenic potential of calcitriol-treated colon carcinoma cells towards stimulatory in a corneal neovascularization assay [132]. Direct transcriptional regulation of VEGF by calcitriol is feasible due to the presence of functional VDREs in its promoter region [136,137]. It is worth mentioning that VEGF induction by calcitriol may help to restore vascular normality, which might be accomplished by restraining pathological angiogenesis and helping to adequately deliver chemotherapy to the tumor mass. Nevertheless, the observation that calcitriol is able to stimulate VEGF or enhance the angiogenic potential of vascular [134,140,141], colon, and breast cancer cells [132,133], together with the reference showing increased serum VEGF levels derived from tumors in calcitriol-treated mice [138], raises prognostic concerns and deserves further studies.

### 3.6. Epigenetic Regulation of the VDR

Epigenetic mechanisms involve modifications of histone proteins (acetylation, methylation, phosphorylation, ubiquitination) and DNA (methylation). Histone acetylation is driven by enzymes called histone acetyl transferases (HATs), and it is associated with gene transcription, opening the chromatin and making it more accessible to transcription factors; while, histone hypoacetylation or deacetylation catalyzed by histone deacetylases (HDACs) activity is associated with gene silencing [142]. The balance of histone acetylation and deacetylation is essential for physiological regulation of gene expression. Imbalance of HATs/HDACs may cause defects in cellular processes such as cell cycle progression, proliferation and survival. Likewise, overexpression of particular HATs and HDACs enzymes takes place in some types of tumors, such as those from the breast, prostate, and colon [143,144,145,146]. In addition, HDACs inhibitors (HDACi), as natural or synthetic compounds whose structure allows them to interact with residues near the active site of HDACs provide another way to modulate the acetylation status of histones. HDACi are known to affect DNA repair mechanisms and chromatin stability. They induce apoptosis in malignant cells [147,148], repress tumor cell proliferation [149], and promote anti-tumor immunity [150].

Activity of VDR can be modulated epigenetically by histone acetylation. After binding calcitriol, VDR forms heterodimers with other nuclear receptors that bind to specific sequences in the promoters of target genes and interact with HATs or HDACs, and recruit co-activators such as RUNX2 [151], for regulation of different genes. RUNX2, a bone-related transcription factor which is cancer-related and ectopically expressed in non-osseous metastatic tumor cells; and is associated with proliferation and motility. The interaction of VDR/RXR with negative VDREs in the DNA of target genes recruits HDACs, which are known to promote gene repression and transcriptional inactivation, thereby reversing HAT activity [152,153].

In prostate cancer cells, calcitriol synergizes the inhibitory effect of an HDACi on target genes associated with the control of proliferation and induction of apoptosis [154], while in non-malignant prostate epithelial cells VDR induced dynamic histone modification patterns at VDR binding sites on the p21 promoter, triggering cell cycle arrest [155]. Calcitriol also regulates p21 transcription in breast cancer cells by inducing cyclical chromatin looping that depends on both histone deacetylation and demethylation [156]. Resistance of cancer cells to calcitriol could be explained by reduced VDR content or by epigenetic mechanisms that selectively suppress anti-proliferative target genes. In this regard, co-treatments with calcitriol plus HDACi resulted in re-expression of anti-proliferative target genes and further synergistic inhibition of proliferation in some cell lines of cancer (breast, prostate, colon and myeloid) [157]. Another HDAC affected by calcitriol is the NAD-dependent deacetylase sirtuin 1 (Sirt 1), a regulator of energy metabolism. VDR binds directly with Sirt1 in a partially ligand-dependent manner and calcitriol enhances the recruitment of Sirt1 to the transcription factor FoxO3a [158] in order to regulate its activation by acetylation and phosphorylation. It is not known if the VDR acts as a scaffold or whether it regulates the enzymatic activity of Sirt1. It appears that VDR is a selective regulator of Sirt1 function [158], but the extent of this regulation in tumorigenesis remains to be clarified. Sirt1 seems to have a dual role in cancer prevention and tumorigenesis, since its down regulation induces the development of multiple tumors in mice, it is overexpressed in different types of cancer [159], its presence in tumors may worsen the prognosis of the disease [160] and treatment with Sirt1 inhibitors has anti-cancer effects [161]. Activation of DNA methyltransferases and Sirt1 is induced by caloric restriction, which leads to changes in the expression of genes such as p53, FoxO, and p16 [162]. Calcitriol supplements can activate the VDR and may reestablish the balance by recruiting Sirt 1 and consequently diminishing pro-inflammatory factors such as NF-κB and enhancing anti-inflammatory factors such as interleukin 10 (IL-10) [158]. Therefore, vitamin D may be involved in the metabolic status of tumors via Sirt1, sensing the redox status of the cell and/or affecting epigenetic regulation. These possibilities need further investigation.

Besides histone acetylation, DNA methylation affects the expression of vitamin D metabolizing enzymes. The expression of the vitamin D hydroxylases and VDR is aberrant in different types of cancer [163]. Indeed, the anabolic enzyme CYP27B1 is downregulated in human prostate tumor [164] and upregulated in colon tumor progression [165], while expression of CYP24A1 is increased in prostate cancer cells [166] and in colorectal tumors [167] and downregulated in breast tumors [168], possibly reducing calcitriol efficacy. The epigenetic modifications are tissue specific [163,169] and their differential effects may explain the lack of response to calcitriol treatment in some types of cancer cells.

### 3.7. Calcitriol Anti-Inflammatory Effects

#### 3.7.1. Inflammation-Dependent Carcinogenesis

Chronic inflammation has been shown to predispose toward tumor development. Indeed, inflammation is causally related to oncogenesis through processes that involve genotoxicity, aberrant tissue repair and/or proliferative responses [170]. Chemical, physical and/or infectious agents such as viruses and bacteria may initiate chronic inflammation through autocrine/paracrine signalization or by influencing the host cell responses [171]. These extrinsic factors may cause non-resolving inflammatory responses that could lead to malignant processes; however, intrinsic factors such as oncogenes or tumor suppressor genes can also be the cause of inflammation-dependent carcinogenesis [172,173]. An example of inflammation-dependent genotoxicity is portrayed by the constant production of ROS by inflamed tissues, which causes DNA damage. In this regard, the initiation of colonic tumorigenesis is thought to be related to a chronically inflamed epithelium acting as a source of ROS and possible reactive nitrogen species [174,175]. This could partially explain why patients with ulcerative colitis and Crohn’s disease are at increased risk for developing colorectal cancer [175]. In addition, other components of the inflammatory reaction also play an important role in inflammation-dependent carcinogenesis; namely, cytokines (both pro- and anti-inflammatory) and immune cells such as tumor-associated macrophages, T-regulatory cells (Tregs,) and B cells. Tumor necrosis factor-α (TNF-α) is the master pro-inflammatory cytokine governing major inflammatory pathways involved in inflammation-induced carcinogenesis, by means of the transcription factors signal transducer and activator of transcription 3 (STAT3) and NF-κB [176]. Both STAT3 and NF-κB signaling may also induce the epithelial-to-mesenchymal transition (EMT) by downregulating the expression of epithelial differentiation markers, a process associated with metastasis [177,178]. EMT may likewise be induced by other cytokines: IL-6 and IL-1 [179,180]. IL-6 and TNF-α can activate STAT3 oncogenic signaling in obesity-dependent carcinogenesis, as seen in HCC [181]. Of note, in diverse human cancerous tumors the persistent activation of STAT3 is associated with tumor progression, which is why this transcription factor has been considered an oncogene [182,183]. Mechanistically, STAT3 induces cell proliferation by upregulating the expression of various cyclins and oncogenes, while it increases cell survival by promoting the expression of the anti-apoptotic genes BCL2 and BCL2-like [184].

#### 3.7.2. Calcitriol Effects upon Inflammation-Dependent Carcinogenesis

Given the oncogenic potential of activated STAT3, the ability of calcitriol to repress signaling through this transcription factor is paramount among its anti-neoplastic effects. In this regard, the constitutive activation of STAT3 has been shown to mediate growth, survival and invasion of breast cancer cells [185], while vitamin D analogs such as Gemini could markedly repress CD44-STAT3 signaling, suggesting its potential to inhibit breast cancer invasion [186]. Moreover, in an *in vivo* and *in vitro* preclinical study of gastric cancer, another non-calcemic analogue of vitamin D, paricalcitol, showed a robust capacity to disrupt inflammation-dependent tumor promotion. Indeed, paricalcitol significantly suppressed the expression of inflammatory mediators such as COX-2, while strongly reducing the levels of phosphorylated STAT3, by limiting the level of NF-κB in the nucleus [187]. Supporting the latter, calcitriol has been shown to intrinsically block NF-κB activity and downregulate NF-κB protein levels in a variety of cell types [188,189,190,191]. A partial mechanistic rationale for the anti-inflammatory effects of calcitriol comprise the stimulation/stabilization of the NF-κB inhibitory protein α (IκBα), the physical interaction of the VDR with IκB kinase β protein (IKKβ) and the blocking of NF-κB binding to DNA, all of which result in NF-κB inhibition [192,193]. These inhibitory effects of calcitriol upon NF-κB are highly relevant for medical oncology, given the critical role that NF-κB plays in cancer pathogenesis and that it is constitutively expressed in several types of malignant tumors [194]. Indeed, NF-κB activation has been shown to regulate the expression of many genes involved in oxidative stress, cellular transformation, proliferation, inflammation, anti-apoptosis, angiogenesis, invasion, metastasis and numerous other potentially carcinogenic processes [194,195,196].

On the other hand, calcitriol has also been shown to affect, in various ways, the synthesis of inflammatory prostaglandins, known for their oncogenic potential. In human prostate cancer cells, for instance, calcitriol repressed the expression of COX-2, the enzyme involved in prostaglandin synthesis, while it upregulated the expression of 15-hydroxyprostaglandin dehydrogenase, which initiates prostaglandin catabolism and secretion. Moreover, calcitriol also repressed prostaglandin receptor expression; thus, the secosteroid affects the prostaglandin signaling pathway at different levels [197]. Noteworthy, calcitriol potently inhibits prominent inflammatory cytokines such as IL-6 and TNF-α in malignant cells as well as in cells under normal and inflammatory conditions [198,199,200,201,202]. The participation of calcitriol in the dialog between cancer and immune cells is well depicted in the article by Bessler and colleagues [202]. In that study, co-incubation of peripheral blood mononuclear cells with colon cancer cells caused a significant stimulation of TNF-α, IL-6 and IL-10 generation by immune cells; however, the addition of calcitriol markedly inhibited secretion of these cytokines [202]. These observations support the beneficial role of calcitriol in suppressing inflammation and subsequently preventing colon cancer. With respect to other types of inflammation-related cancers, it has been observed that TNF-α production by ovarian cancer cells stimulates a network of other inflammatory cytokines and angiogenic factors that seem to act autocrinally/paracrinally to promote colonization of the peritoneum and neovascularization of the incipient tumors [203]. The fact that TNF-α is a target of calcitriol warrants further research on calcitriol in ovarian cancer. On the other hand, the incubation of human myelomonocytic U937 leukemia cells in the presence of calcitriol and retinoids resulted in the accumulation of transforming growth factor beta (TGF-β) in the culture media. TGF-β is known to suppress inflammation, inhibit proliferation and promote differentiation at early stages of oncogenesis, and its induction by calcitriol caused cell differentiation and inhibition of cell proliferation [204]. Calcitriol also induced the expression of prostate-derived factor (PDF), the pro-apoptotic protein of the TGF-β superfamily, in androgen-responsive prostate cancer cells through a VDR/p53-dependent process [205].

A well-recognized effect of calcitriol is to promote innate antimicrobial defense mechanisms, including up-regulating antimicrobial peptides such as cathelicidin and DEFB4 [206,207]. The latter strongly suggests that another mechanism of calcitriol used to prevent carcinogenic processes related to chronic inflammation is to abate the infectious agents such as viruses and bacteria that could be involved in the initiation or perpetuation of this process. This, together with the inhibitory actions of calcitriol upon inflammatory cytokines, explains the considerable recent interest in exploring vitamin D as a supplementary immunomodulatory therapy in the treatment of infectious diseases associated with chronic inflammation. Indeed, calcitriol can promote resolution of chronic inflammation, helping to prevent its potential carcinogenic effects. Immunosuppressive factors are needed in order to act as negative feedback agents and restrain any potential exacerbated immune reaction that could be detrimental for the patients’ health. Nevertheless, as previously discussed by Balkwill and collegues [173], inflammation may not always be “bad” in the context of malignant disease. Ultimately, inflammation is there for a reason; a natural reaction of the body to either exogenous or endogenous insults. In this regard, the immune system is able to recognize and destroy neoplastic cells, preventing tumor progression. Interestingly, this property may be targeted strategically by tumors in order to evade the host immune surveillance. Indeed, tumor-induced immunosuppression is partially mediated by anti-inflammatory cytokines such as IL-10 and TGF-β produced by the transformed cells. Even though these two cytokines may be anti-oncogenic in early stages of the disease (during chronic inflammatory insults), they may become detrimental to the host by promoting progression of advanced/established tumors [208]. Certainly, IL-10 and TGF-β may help tumors evade immune surveillance, with the consequent progression and dissemination. For example, IL-10 promotes lung cancer growth by suppressing both T cell and antigen-presenting cell function [209], while tumoral TGF-β production in human HCC evokes pro-tumorigenic and dissemination effects by a crosstalk with chemokine receptor type 4 (CXCR4) and its ligand, stromal cell-derived factor 1. This dual role of immunosuppressive molecules in the context of cancer raises the question of whether calcitriol, as an additional immunosuppresor, might behave similarly. In the end, it might all depend on the context (microenvironment) and tumor particularities, such as the disease stage (advanced *vs.* early stages), the molecular signature of the tumor and its location. In addition, the *in situ* concentration of the hormone/cytokine and the equilibrium of pro- and anti-oncogenic factors are also determinants of the tumor’s fate. Indeed, that deserves to be further investigated. However, it should be taken into consideration that calcitriol acts by a variety of mechanisms of action to suppress cancer growth, such as those described in this and other reviews; therefore, the final biological outcome is expected to be anti-neoplastic, as widely described in the literature.

## 4. Conclusions

The identification of calcitriol effects that do not involve mineral homeostasis highlights a role for this hormone beyond bone health. Non-classical effects of calcitriol through the VDR have been discovered in many tissues and cells outside the kidney, where its biosynthesis may also take place, e.g., in placenta, pancreas, breast, prostate, and immune cells. Vitamin D deficiency observed in patients with a variety of cancers drove the interest to find its pathophysiological significance and to evaluate the effect of vitamin D supplementation on the course of these diseases. To this date, vitamin D research seeks to understand the mechanisms through which calcitriol acts and to explore its therapeutic potential in the oncology field. Even though vitamin D stands as a promising safe alternative for cancer prevention and progression to a reasonable extent, an adequate dietary cholecalciferol intervention strategy still waits to be correctly established. Likewise, the use of the active vitamin D compound calcitriol as a therapeutic agent is also under intense research. Indeed, more information is needed in order to know the minimum quantity required to bring about a clear effect on cancer markers as well as the ideal time for intervention. Of primordial importance is to properly understand the overall biological effect of calcitriol, taking into consideration that it acts differently depending on the type of cancer and use of other drugs. In this review we have discussed the complex interplay of the processes started by calcitriol in cells and *in vivo* models of different types of cancer comprising themes such as angiogenesis, regulation of kinases cascades, and inflammation. In particular, the dual nature of calcitriol (suppressor of active immunity, inducer of innate immunity) makes it an appealing compound to treat and prevent inflammation-dependent carcinogenic processes given that it can promote resolution of chronic inflammation and infection, which deserves further investigation.
